# Yoga, a mindfulness therapy to prevent PTSD as to encompass athletes’ performance

**DOI:** 10.3389/fpsyg.2024.1334278

**Published:** 2024-04-23

**Authors:** Rocsana Bucea-Manea-Țoniș, Dan Gh. Păun

**Affiliations:** ^1^National University of Physical Education and Sports, Bucharest, Romania; ^2^Department of Physical Education and Sports, Spiru Haret University, Bucharest, Romania

**Keywords:** yoga practice, medical rehabilitation sports training, technology, post-traumatic stress disorder, therapy

## Abstract

**Introduction:**

Yoga is one of the physical and mental activities used in elite sports training for risk prevention and medical rehabilitation in case of injuries caused by overtraining or accidents. This study examined the opinions of Romanian elite athletes and coaches on the feasibility of incorporating yoga practice into training regimens for purposes of injury prevention and medical recovery.

**Methods:**

This study surveyed a group of 500 athletes, coaches, and medical personnel from three universities in Romania, all of which are part of the Faculty of Physical Education and Sport (PES). An online survey was administered which evaluates athletes’ experience of yoga integration in pre/post training and its positive effects on reducing posttraumatic stress disorder (PTSD). The data were then analyzed with a structural equation model utilizing SmartPLS software.

**Results:**

According to the survey, Romanian athletes use yoga both before and after competitions to improve their focus, balance, muscle, and joint elasticity, foster a winning mindset, control their emotions and PTSD, visualize their competition performance, and see themselves as winners. The survey also found that yoga is seen as useful for cardiac rehabilitation, neuropathic pain, pulmonary disease, orthopedic illness, muscle strain, and managing symptoms of Posttraumatic Stress Disorder (PTSD).

**Conclusion:**

This study contributes to enhancing athletes’ mindfulness and health, offering valuable insights to trainers and athletes interested in incorporating yoga into professional sports activity. The results support the notion that yoga integration in training activity promises to positively influence athletes’ performance and reduce collateral side effects of competitions. The results are also in line with the objectives of the Global Action Plan on Physical Activity 2018–2030 (GAPPPA) - with the theme of “being more efficient to prevent than to treat” – which places special emphasis on the demands for certain programs and services, sports coverage, and healthy workplace initiatives. The study further indicates that Romanian elite athletes and coaches support the use of yoga is an effective method for enhancing athletic training and medical therapy for post-traumatic illnesses and stress disorders.

## 1 Introduction

Yoga practice has been increasingly used in sports training and as a therapeutic method, including for individuals with post-traumatic stress disorder (PTSD). In recent years, technology has also played a role in the development and implementation of yoga-based interventions for these purposes. In sports training, yoga practice can help to improve flexibility, balance, strength, and mental focus, all of which can contribute to better athletic performance. By incorporating yoga into their training routines, athletes may be able to reduce the risk of injury and achieve better results in their sport. Yoga also helps the implementation of the Global Action Plan on Physical Activity 2018–2030: More Active People for a Healthier World (GAPPA, 2018). GAPAA places special emphasis on the demands for certain programs and services, sports coverage, and workplace initiatives (Taylor et al., 2021). The five most popular forms of exercise—aerobics, walking, practicing yoga, Zumba, and jogging—all significantly lower the wellness risks linked to inactivity (Aman et al., 2022). For example, exercise like yoga can improve balance and selective attention, aiding elderly individuals in cognitive and motor abilities, and thereby enhancing their well-being (Taheri et al., 2018). A considerable improvement in flexibility, lower extremity muscular power, and static and dynamic balance was obtained with Pilates and yoga workouts (Irandoust and Taheri 2016).

Athletes have to face overtraining, the stress of the competition, and sometimes PTSD. These facts were more frequent after the long period of partial inactivity due to COVID-19. Many coaches are looking for alternative solutions to prevent these consequences. As some authors proved that yoga training positively influences the prevention and recovery of PTSD, we decided to find out the opinion of Romanian athletes.

Athletes who utilize yoga to prevent PTSD become role models and brands, and they have the power to inspire anyone to practice yoga in their free time. Yoga offers several benefits, including the ability to be practiced by anybody, virtually anywhere, without the need for equipment or technology, as long as they are aware of their limitations. Thus, yoga helps in reaching the GAPPA objective.

This research analyzes the perceived advantages brought by yoga to Romanian athletes’ mental and physical training. It also researches the modalities that help Romanian athletes to cope with PTSD by means of yoga.

## 2 Literature review

Numerous studies have shown that yoga has extra benefits for reducing stress and treating and preventing chronic non-communicable diseases. During the COVID-19 pandemic’s seclusion and quarantine periods, technology, the Internet, and yoga exercises were the major sources of psychological uprightness, social connections, academic training, and employment (Mindrescu and Enoiu, 2020).

The metamorphosis of elite athletes requires the development of physical endurance and flexibility in addition to handling stress, resiliency, tranquility, mind–body awareness, and spiritual/personal growth. The effectiveness of the global health system has a positive influence on these processes, notably in terms of both mental and physical well-being (Cheshire and Cartwright, 2021).

### 2.1 Yoga encompasses athletes’ performance and mental health

The top athlete can develop better self-awareness and self-regulatory skills compared to non-practitioners through the regular and proper practice of yoga postures and meditation, which is reflected in greater levels of interoceptive awareness and decentering abilities (Vago and Silbersweig, 2012; Tolbaños-Roche and Menon, 2021). Yoga lessons have a deliberate inner focus on awareness of the self, breath, and energy, as well as physical activity (Iyengar, 2021). Yoga practice produces a physiological condition that reverses the stress reaction, which makes it possible to establish harmony and oneness between the body and mind. Yoga is utilized in sports to achieve optimum physical fitness and avoid injuries while enhancing performance. A yoga intervention improves athletes’ flexibility, muscular strength, endurance, and cardiovascular performance, according to certain research (Polsgrove et al., 2016; Laishram et al., 2022), as well as physiological health markers including heart rate, immunological function, diastolic blood pressure, muscle pain, and mental fitness. Within 6 weeks, people can feel the effects of yoga on their flexibility, even if they just practice once per week. In yoga training can result in significant gains in respiratory muscle strength and endurance. Yoga can significantly reduce the amount of body fat in female students and enhance cardiovascular fitness, abdominal muscular endurance and strength, and mobility (Shires, 2021; Subhra et al., 2021).

Yoga is a practice that athletes use in their training regimens to improve performance. Attention, emotion, and yoga-inspired aspects can improve performance when combined with intellectual, metacognitive, and procedure management techniques (Popa et al., 2020). Numerous factors, including load, volume, frequency, mental-physical link, contraction speed, work-rest ratio, and amount of isometric exercise duration, affect how the muscle adapts to high-level training (Petre et al., 2022).

### 2.2 Yoga as a cardio-respiratory therapy

Based on the fact that yoga enhances respiratory and immune system functioning, several authors have created relatively simple-to-follow integrated yoga modules in the form of video recordings to be practiced for illness prevention (Nagarathna et al., 2020; Sawant et al., 2021).

Due to superior intercostal muscular control brought on by greater muscle endurance from yoga practice, persons who seemed to be in good health and were given a 12-week yoga workout regimen had a substantial improvement in VO2 max. Positive effects were observed on the athletes’ physiques and endurance measurements (Lau et al., 2015).

Male football players’ pulmonary function and VO2 max significantly improved after using yogic breathing techniques. Football players’ pulmonary functions are improved when they regularly use yogic breathing techniques in addition to their training (Jangam et al., 2022). Football players who practice yoga and self-myofascial release techniques notice a considerable increase in their flexibility and explosive strength in their legs (Laishram et al., 2022).

Exercise therapies that included yoga, aerobic interval training, and exercise-based cardiac rehab (CR) improved the ability to exercise and lessened the burden of atrial fibrillation (Elkhair, A., et al., 2023). Another study evaluated the impact of Hatha yoga classes on the cardiovascular hemodynamic variables and physical capability of patients with ST-elevation myocardial infarction (STEMI) who were enrolled in a traditional heart rehabilitation program. The enhancement of left ventricular end-diastolic diameter left ventricle end-systolic diameter, and heart rate over time are the most noticeable adjustments to echocardiography parameters and physical capability. The results showed that the CR routine with a revised Hatha yoga training regimen was more effective. Training in Hatha yoga can be suggested as an addition to conventional CR (Grabara et al., 2020).

### 2.3 Yoga prevents PTSD

The number of injuries among athletes is pandemic. The cognitive and physical strains placed on athletes by psychosocial stresses and training regimens significantly enhance their risk of injury. Mindfulness practices are the main focus of all therapeutic programs and therapies for stress reduction and the treatment of different stress- and lifestyle-related health conditions. Effective self-regulation techniques are a key component of the yoga path to obtaining holistic health and well-being. Muscle stretches, isometric and aerobic exercise, stability training, and mental-body activities (such as Tai Chi and yoga) were all included in the agreement of experts’ recommendations for exercise (Zhang et al., 2021).

Yoga can promote the ability of an intervention to lower two critical risk factors for injury: generalized tiredness as well as perceived susceptibility to injury. Yoga can be effectively integrated into players’ sports regimens. Sports including soccer, handball, swimming, winter sports, and baseball are examples that may profit from yoga PTSD (Laux et al., 2015; Hilton et al., 2019; Arbo et al., 2020).

All athletes can benefit from yoga, but it is particularly effective in preventing injuries in sports like running, basketball, tennis, and baseball, football which call for fast movements. By increasing vagal (parasympathetic) engagement and reducing the sympathetic nervous system’s and the hypothalamic–pituitary–adrenal axis’s stress response, yoga is hypothesized to have therapeutic effects. Yoga closely mimics athletic performance in terms of balance, flexibility, strength, endurance of muscles, and efficient movement (coordination) (Jeitler et al., 2020; Shires, 2021; Subhra et al., 2021; Laishram et al., 2022).

Yoga is frequently practiced by athletes as part of their training to improve their physiological capabilities, performance, and self-control while lowering their risk of developing PTSD. Target participants’ memory as well as cognition are improved by yoga. This is because yoga can benefit athletes who experience high levels of tension. Increased social–emotional competency will be shown as a result of yoga practice over time (Shires, 2021).

The consequence will be an overall improvement in mental as well as physical wellness (Molina et al., 2020). Yoga may be included in injury-prevention plans for several reasons, including greater relaxation, increased core stability, and increased mobility and range of motion. According to several researchers, practicing yoga for 4 weeks led to a substantial decrease in weight loss and a rise in the experimental group’s leg and back strength. In a study of handball players conducted by Godara et al., it was discovered yoga practice significantly boosted the players’ back strength (Subhra et al., 2021; Bucea-Manea-Țoniș et al., 2023). A significant increase in the strength of the legs was observed in the research with a structured training program that included yoga teaching saw a substantial improvement in following the intervention agility and flexibility assessments as well as a dramatic improvement in balance after intragroup therapy (Kim et al., 2015; Donald et al., 2018).

Overall, the reviewed research highlighted several benefits of adding yoga to the medical rehabilitation and injury prevention of athletes (Ferradás-Galloso et al., 2022).

Considering into account all of these factors, the goals of the current study were to determine and comprehend if Romanian athletes including their coaches were aware of the advantages of practicing yoga as well as the extent to which they did so. We decided to create a questionnaire to evaluate the current situation in Romania using the knowledge we had acquired. We also looked at how they felt about the connection between yogic asanas, breathing, and top training. The study also examined their opinions on the value of using yogic asanas to lessen post-traumatic stress disorder. We also sought the perspectives of athletes and instructors since we think it is essential to develop more outreach programs and offer doable, relevant, and affordable chances to support yoga participation in medical rehabilitation. We created an online poll for top athletes and their coaches to accomplish our aim. We designed a structural equation modeling, also known as SEM factor analysis, after gathering and selecting the data, to determine the relative relevance of each element.

## 3 Materials and methods

### 3.1 Preliminary phase of the research

Yoga, sports, and physiotherapy (Topic), 2019–2023 (Year of Publication), breath (All Fields), technology (All Fields), articles or review articles (Document Types), and all Web of Science Categories were used to define the research design. We reviewed numerous articles on the benefits of yoga for injury prevention and the relationship between yoga practice and medical recovery in sports. Yoga does not have an extensive background in Romania. As a result, we decided to investigate the extent of awareness about this issue and its possible influence on elite athletes. In detail, we investigated the opinion of elite athletes and coaches regarding the integration of yoga asanas in sportive training to reduce PTSD associated with high levels of competition and overtraining.

The study looks into how yoga may help with sports performance as an adjunct way to providing the best possible sustainable system of including yoga in athletes’ training, and medical rehabilitation.

The main hypothesis of the research is:

H1: The integration of yoga sessions in pre-post athletes’ training will reduce side effects of competitions and overtraining, such as stress, anxiety, and post-traumatic stress disorder.

### 3.2 Design and research phase

The survey (https://forms.gle/FrbqKHCeXaNx9VfT8, available online) was administered to carefully selected teachers and athletes from three universities in Romania, all of which are part of the Faculty of Physical Education and Sport (PES). There were about 500 athletes, coaches, and medical personnel from the university in attendance. To be included in the study, participants had to meet certain criteria, including being athletes, having experienced yoga practice, and being informed of the safety rules of practicing asanas. The study took place from 15 May 2023 to 15 October 2023.

To protect the rights and welfare of the subjects, ethical issues were paramount throughout the whole study. All participants gave their informed permission before the start of the study, guaranteeing that they had all the details about the goal and methods of the investigation and that they had the option of opting out at any moment. To preserve participant privacy, the researchers also upheld stringent secrecy and data anonymity. The study demonstrated the researchers’ dedication to putting the participants’ rights and well-being first at every level of the investigation by closely adhering to ethical criteria for research engaging human subjects.

Twenty of the 238 responses we received were invalidated as being missing or having inconsistent answers. We complied with this condition because the threshold for an appropriate sample is 218. The results cannot be extrapolated to the total Romanian athlete community, but this preliminary investigation will allow and launch additional studies involving in-depth specific issues. The use of yoga in sports (pre−/post-training), the methods for incorporating yoga into professional sports, or how yoga works for dealing with anxiety, tension, or post-traumatic stress disorder (PTSD) because of the intensity of competition and excessive training in sports have all gotten responses from trainers, teachers, and athletes. The majority of the questionnaire’s closed-ended inquiries included numerous viable answers or narrowly defined possibilities. It was conducted online using Google Forms with participants’ permission to handle their replies following GDPR guidelines. Additionally, we offered open-ended inquiries to start the qualitative investigation.

The questionnaire was guided by other investigations, such as those put forth by various authors (Ross et al., 2013; Cramer et al., 2019; Cartwright et al., 2020), as well as by the main author’s expertise as an instructor in yoga, by teachers and athletes that employed sequences of various asanas in their training for professional sports and by a physiotherapist that applied yoga as adjuvant therapy in sports rehabilitation.

At this point, both the theoretical and the practical variables had been established, and scales had been chosen for assessing them, defining the knowledge-gathering approach, selecting the data acquisition device, and constructing the methodological framework to organize the information. The data were evaluated using the partial least squares (PLS) structural equation modeling technique, which analyzes simultaneous interactions among latent, formative, or reflecting variables even for smaller samples. Two latent reflective constructs (Yoga Training and PTSD) are present in our model. All variables, with their descriptive items, are presented in Table 1. In this table, each item represents a question of the survey. The first 10 questions (balance, …, pulmonary) formed the variable Yoga Training and the last 4 questions (competition, … unrest) formed the PTSD variable. The impact of each item on yoga training or PTSD might be measured by loading factors presented in Table 1, which have high values, closer to the maximum point =1. The qualitative feedback was analyzed through open questions where athletes could freely express their opinions.

**Table 1:** Variable description, loading factors, and Variance inflation factor (VIF).

Variables	Items	Description	Loading factors	Collinearity
Yoga training	Yoga is practiced in sports (pre/ post training) to		LF	VIF
	Balance	Improve physical and mental balance	0.70	3.21
	BMI	Weight control assessed by body mass index (BMI)	0.91	4.98
	Cardio	Improve cardiovascular performance (heart rate, diastolic blood pressure)	0.79	4.42
	Control	Improving self-control and mental focus	0.89	4.41
	Endurance	Achieve performance by increasing muscle strength and endurance	0.70	3.08
	Fitness	Achieve performance by reaching the maximum level of physical fitness	0.84	3.07
	Immunity	Improve the immune system	0.77	3.38
	Mobility	Achieve performance by increasing joint mobility and muscle elasticity	0.89	3.32
	Prevent	Prevent injuries by preparing physically and mentally for the competition	0.80	2.42
	Pulmonary	Increase lung capacity and strengthen the diaphragm muscle	0.92	5.00
PTSD	Yoga reduces stress, anxiety, and post-traumatic stress disorder			
	Competition	Due to competition	0.88	3.08
	Daily	Involvement in too many daily activities	0.92	3.46
	Overtraining	Reduces post-traumatic stress disorder due to over-training	0.84	4.87
	Unrest	Lack of rest	0.88	4.05

Our analysis contains an SEM confirmatory factor analysis using SmartPLS. In the field of structural equation modeling (SEM), statistical methods like path analysis are frequently employed to examine intricate correlations between variables.

## 4 Results

The frequency, the association of variables, graphical images, and table were created using the examined data, and the SmartPLS tool also created a qualitative study regression model.

We used Path Analysis because it: (a) lowers the variance of endogenous construct residuals; (b) has minimal detection concerns; (c) yields relevant findings regardless of tiny sample sizes; and (d) predominantly blends formative and reflective components, SmartPLS is a trustworthy regression approach (Chin et al., 2010).

Whenever the structural model becomes exceedingly complicated, the sample is quite small, and the hypothesis contains both formative and reflective elements, PLS-SEM (partial least squares SEM) or path analysis is recommended to be utilized. When a study involves developing theories or forecasting (with contributions to theory development), PLS-SEM is the optimal approach. Its primary applications are in predictive analysis and the clarification of complicated linkages (Hair et al., 2022).

We examine if the survey’s questions help establish the relevance of a claim or hypothesis to assure the reliability of the survey. A survey is considered to be trustworthy and reliable when all of its questions and results match up (overall score). The coefficient of Cronbach’s alpha index rises with the number of items (questions).

It is recursive in nature and chooses items according to how they contribute to the overall score after evaluating how each item’s relationship to each other and the final score is related to that of the other items. The main criterion for this process is Cronbach’s alpha index, which must have a value between 0 and 1. For consistency, a scale ought to be as near to 1 as feasible, with 0.70 being usually accepted as the highest limit.

### 4.1 Construct reliability and validity

**Figure 1:**
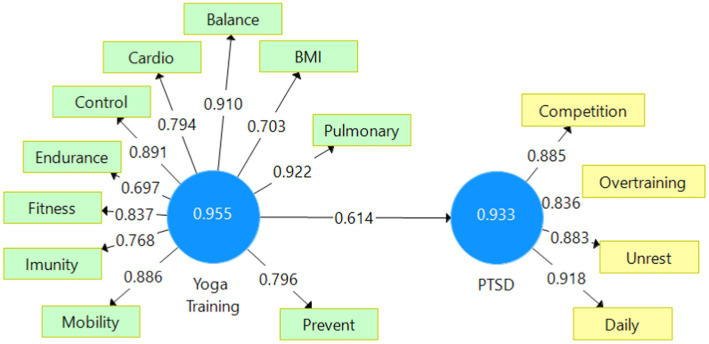
Cronbach’s alpha analysis and Path coefficients. Source: SmartPLS analysis (reprinted from a free version of SmartPLS software, version 3.3.9, created on 11 March 2023) (Hair et al., 2022).

The study made use of SmartPLS (Henseler et al., 2015), as shown in Table 2, to evaluate consistency using composite reliability. The permitted acceptable thresholds for a reliable model include composite reliability (>0.6), Cronbach’s alpha, rho_A (>0.7), and average variance extracted (AVE > 0.5). The survey items are suitable for our research, as they are based on Cronbach’s alpha coefficients, which suggests that our assumption is supported. For each criterion, all of our variables display high values (Table 2). The coefficient of determination or R-squared claims that the variance of Yoga Training explains 37.8% of the variance of the reduced PTSD symptoms (Table 2 and Figure 1).

**Table 2:** Model validation criteria.

Reflective constructs	Composite reliability	Cronbach’s alpha	AVE	rho_A	R- Squared
	(>0.7)	(>0.7)	(>0.5)	(>0.5)	(>0.5)
PTSD	0.933	0.933	0.776	0.934	0.378
Yoga Training	0.955	0.955	0.679	0.958	

Source: SmartPLS analysis (reprinted from a free version of SmartPLS software, version 3.3.9, created on 2 Nov 2023) (Hair et al., 2022).

The latent variable correlation shows a rather strong positive correlation (0.614) between Yoga Training and PTSD. The path coefficient has the same value (0.614) (Figure 1), meaning that H1 is accepted: The athletes that had integrated yoga asanas in pre/post training have reduced the side effects of competition and overtraining.

### 4.2 Discriminant validity

The calculation of discriminant validity was performed. It is defined as the extent to which a variable in the structural model empirically differs from other variables. Given that the Fornell-Larcker Criterion is satisfied, our model has statistical power, because its values are less than the. It indicates that, when scales are compared two at a time, discriminant validity is probably present (Table 3).

**Table 3:** Discriminant validity.

Criterion	Fornell-Larcker		HTMT	
Variables	PTSD	Yoga training	PTSD	Yoga training
PTSD	0.88			
Yoga training	0.61	0.82	0.61	

Source: SmartPLS analysis (reprinted from a free version of SmartPLS software, version 3.3.9, created on 27 June 2023) (Hair et al., 2022).

The model is statistically robust as Heterotrait–Monotrait (HTMT) criteria are met. HTMT ratios should be <0.85 to achieve discriminant validity (Henseler et al., 2015), meaning that all constructs were statistically differentiated from each other when taken two by two (Table 3).

### 4.3 Model fit

Model fit was assessed using approximate fit indices such as standardized root mean square residual. In our case, SRMR has a value of 0.063, which is very close to 0.05. (Table 4). The model is better the higher the NFI values (the closer to 1). Consequently, we may state that our model fits and that the H1 hypothesis is accepted (Wu and Estabrook, 2016).

**Table 4:** Fit summary.

	Saturated model	Estimated model
SRMR	0.063	0.063
NFI	0.849	0.849

Source: SmartPLS analysis (reprinted from a free version of SmartPLS software, version 3.3.9, created on 2 Nov 2023) (Henseler et al., 2015).

### 4.4 Multicollinearity analysis

To evaluate the importance of variables, the SmartPLS algorithm computed the variance inflation factor (VIF) of each construct. Table 2 provides a summary of the findings. Given that there are no VIF values greater than 5, there is no multicollinearity between the variables (Reinartz et al., 2009).

**Figure 2:**
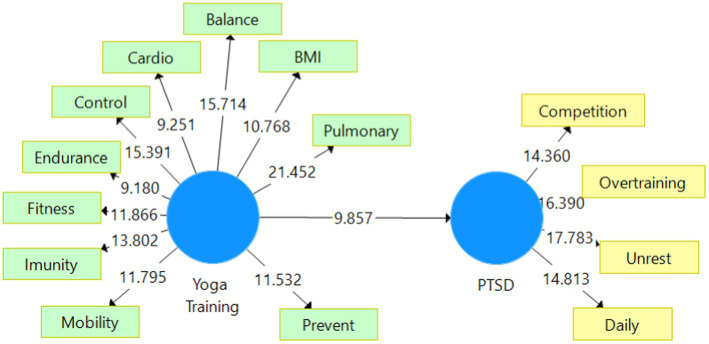
Bootstrapping. Source: SmartPLS analysis (reprinted from a free version of SmartPLS software, version 3.3.9, created on 10 June 2023) (Hair et al., 2022).

The *p*-values for each of the five SEM regressions are all less than the 0.05 cutoff, demonstrating once more the strength of our models’ design (Table 5). The t-test data are indicative. Figure 2 gives a summary of the results. Two-tailed t-tests had a bootstrapping value greater than 1.96.

**Table 5:** Bootstapping *t* test statistics.

Direct effect	Original sample	Sample mean	Std dev	*t* test statistics	*p* values
Yoga training ➔PTSD	0.614	0.621	0.062	9.8	0

In Table 5 we may observe a direct effect, too (Yoga Training ➔PTSD), with an important weight. It highlights that the more practicing yoga in pre/post training by the athletes, the better their results in reducing PTSD.

After taking into account all of the validation steps shown in Tables 2–5 and Figures 1–2, we may assume that the construct indicators are strongly positively associated, and this assumption H1 has been approved. We can therefore conclude that the higher the degree of knowledge regarding yoga, the higher the inclinations of athletes, and coaches’ inclination of using therapy asanas in sports training and medical rehabilitation. Yoga was used in sports by Romanian athletes for the treatment of PTSD due to competition and overtraining, to reduce mental tension or to energize the body, to treat muscular stiffness, to set and visualize goals, to increase cardio-respiratory functions or treat associated illness, in other posttraumatic conditions such neuro-muscular, neuro-mental, immune diseases, etc.

## 5 Discussion

Romanian athletes have learned a great deal about yoga. They believe that yoga has become a part of health maintenance since it is practiced frequently while controlling one’s breathing and is taught by certified instructors in Romanian sports clubs. They equated yoga with a basic and therapeutic preventative measure and an Asian school of thinking and philosophy that seeks to harmonize body, mind, and spirit. Other authors found the same results (Lau et al., 2015; Subhra et al., 2021; Jangam et al., 2022; Bucea-Manea-Țoniș et al., 2023).

These specialized skills led them to engage in yoga therapy asanas to achieve balance, physical and mental relaxation, pain relief, ligament and muscle stretching, mental focus and endurance under anaerobic conditions, postural correction, spine stress relief, muscle toning, increased respiratory frequency and capacity through deep breathing, lymphatic drainage, and endocrine system modulation, blood circulation stimulation, and pulse regulation, in the opinion of Romanian athletes. Other authors found the same results (Jeitler et al., 2020; Shires, 2021; Subhra et al., 2021; Laishram et al., 2022; Bucea-Manea-Țoniș et al., 2023).

Their understanding of yoga led Romanian athletes to use it for primary prophylaxis, as a therapeutic approach (can treat respiratory, circulatory, neuro-muscular, neuro-mental, and immune diseases, etc.), for fitness and stress reduction, to improve physical and mental balance, as well as for stress reduction. They chose yoga because it eradicates cultural, gender, and ethnic discrimination and cultivates a positive outlook in the face of success and failure. Authors like Polsgrove et al., 2016, Mindrescu and Enoiu, 2020, Cheshire and Cartwright, 2021 reached the same results.

Before games (football, polo, volleyball, basketball, handball, etc.) or individual sporting events, one can perform breathing techniques from yoga to relieve mental strain or to energize the entire body, as Romanian athletes affirm. Yoga is practiced by athletes to minimize post-traumatic stress disorder due to competition and overtraining. Yoga is used to improve the triple extensor chain, which aids in proper movement execution in group or individual sports, and to restore the athlete’s homeostasis following a match or competition through breathing exercises. Additionally, yoga improves mental health by lowering stress, anxiety, sadness, and post-traumatic stress disorder (PTSD), favoring the mental state of competition. Other authors found the same results (Laux et al., 2015; Hilton et al., 2019; Arbo et al., 2020; Popa et al., 2020; Petre et al., 2022; Elkhair, A., et al., 2023).

Yoga is a beneficial practice for physical, mental, and emotional well-being, but it has limitations and may not be suitable in every context and for anyone. Physical limitations may make certain poses challenging or risky for those with specific physical issues, injuries, or medical conditions (Wiese et al., 2019). Advanced yoga poses require significant skill, strength, and flexibility, which beginners or those with limited physical abilities may find inaccessible, but for athletes has a positive influence on their training and reducing PTSD. Emotional and mental health concerns may arise, and those with severe mental health issues should seek professional help. Cultural appropriation concerns arise from the practice’s cultural roots in Hinduism and other Indian traditions. Individual variability is another issue, and yoga requires time and commitment to see benefits. It may not be the most effective form of exercise for certain fitness goals, such as building muscle mass or improving cardiovascular endurance. Therefore, it is crucial for individuals to approach yoga mindfully, listen to their bodies, and consult with healthcare professionals. In our research, we focused on the advantages of yoga practice, as we are surveying professionals in sports who know how to avoid these risks.

## 6 Conclusion

Yoga is commonly used in sports for the treatment of PTSD caused by competition and excessive exercise, to alleviate mental stress or to energize the organism, to manage muscular rigidity, to set and envision targets, to boost cardio-respiratory functions or to manage associated illness, and in other posttraumatic ailments such as wrist tunnel syndrome, neuro-muscular, neuro-mental, immune-related conditions, and so on.

The goal of this article was to find out the level of acquaintance of Romanian performance athletes and coaches. Through a survey, we screened the level of integration of yoga therapy asanas in performance sports training and rehabilitation. We found out that the greater the level of information about yoga, the more players, coaches, and medical personnel are inclined to use treatment asanas in sports training and physiotherapy. This high level of information allows participants to better appreciate the advantages of yoga.

There are several limitations to our investigation. The study may be reproduced in different nations and with different samples, even though the sample size is typical for our statistical population, in order to compare the outcomes and gain a better understanding of the yoga advantages and disadvantages if included in training. The model is another drawback. Future study is advised to examine the interaction between several variables, including the extent of information about the benefits and drawbacks of yoga, the degree to which yoga is included in training, and the effect on athletes’ performances, in order to construct more assertive models. Additionally, our study’s rigorous literature review criteria should be expanded upon. We have not surveyed any articles that aren’t in WoS yet.

## Data availability statement

The raw data supporting the conclusions of this article will be made available by the authors, without undue reservation.

## Ethics statement

The studies involving humans were approved by Ethics Committee of Research of Transilvania University of Brasov, Faculty of Physical Education and Mountain Sports, Romania. The studies were conducted in accordance with the local legislation and institutional requirements. The participants provided their written informed consent to participate in this study.

## Author contributions

RB-M: Writing – review & editing, Writing – original draft, Visualization, Validation, Supervision, Software, Resources, Project administration, Methodology, Investigation, Formal analysis, Data curation, Conceptualization. DP: Resources, Writing – review & editing, Data curation.
